# Artificial Intelligence-Based Muscle Ultrasound: A Novel Approach to Nutritional Evaluation Beyond Quantity in Neurological Patients

**DOI:** 10.3390/nu18162676

**Published:** 2026-08-16

**Authors:** Juan José López-Gómez, Lucía Estévez-Asensio, Elena Santos-Pascual, Olatz Izaola-Jauregui, Paloma Pérez López, Ángela Cebriá, Beatriz Ramos-Bachiller, Eva López-Andrés, Mario Alfredo Vasquez-Saavedra, David Primo-Martín, Daniel Rico-Bargues, Eduardo Jorge Godoy, Daniel A. de Luis-Román

**Affiliations:** 1 Servicio de Endocrinología y Nutrición, Hospital Clínico Universitario de Valladolid, 47003 Valladolid, Spain; 2 Centro de Investigación en Endocrinología y Nutrición, Universidad de Valladolid, 47005 Valladolid, Spain; 3 Health Research Institute of Valladolid (IBioVALL), 47010 Valladolid, Spain; 4 DAWAKO Medtech SL, Parc Cientific de la Universitat de Valencia, 46980 Paterna, Spain; 5 Técnicas Avanzadas de Desarrollo de Software Centrado en la Persona, Departamento de Informática, Universitat de Valencia, 46100 Burjassot, Spain

**Keywords:** neuromuscular disease, artificial intelligence, disease-related malnutrition, sarcopenia, muscle ultrasound

## Abstract

Background: Neurological disease may lead to malnutrition through disease-related complications, underscoring the need for accurate muscle assessment. This study aims to evaluate an AI-based tool for quantifying and characterizing muscle ultrasound images, comparing its performance with the usual techniques of muscle mass and function. Methods: This was a prospective, open-label, longitudinal observational study of 117 adults with neurological disorders at high nutritional risk, designed to evaluate nutritional status and clinical evolution. The clinical assessment integrated anthropometry, bioelectrical impedanciometry, handgrip strength, dysphagia testing, and rectus femoris quadriceps ultrasound. Ultrasound images were evaluated through an AI-based platform to extract muscle quantity (rectus femoris muscle area (RFMA) and rectus femoris muscle thickness (RFMT) and quality biomarkers (percentage of low-echogenicity areas (Mi), interpreted as muscle; percentage of medium-echogenicity areas (FATi), interpreted as intramuscular fat). Patients were followed for two years to record mortality. Results: The sample included 117 adults with neurological disorders (52.1% women), with a mean age of 63.01 (16.14) years. A total of 77 patients (65.8%) had a condition with direct neuromuscular involvement. According to Global Leadership Initiative on Malnutrition (GLIM) criteria, 73 patients (62.4%) had malnutrition, while 30 patients (25.6%) had severe malnutrition. There were no differences in muscle mass parameters, but patients with neuromuscular involvement (NM) had lower values of percentage of Mi (NM: 42.44 (9.09%) vs. 47.36 (7.74)%; *p* < 0.01), and higher values of FATi (41.91 (5.71)% vs. 39.15 (4.89)%). The prevalence of mortality was 26 patients (22.2%). In the multivariate analysis, FATi (above median) (OR = 5.11 (IC95%: 1.26–20.67)) increased risk of death, adjusted by age, neuromuscular involvement, sex, and Mi. Conclusions: Patients with neuromuscular disorders showed a markedly lower proportion of Mi and a higher presence of FATi compared to those with non-neuromuscular conditions. Mortality was associated with greater FATi on AI-based ultrasound analysis. These findings suggest AI-enhanced imaging captures clinically relevant tissue alterations with potential prognostic value; however, given the observational data and heterogeneity of neurological conditions, these implications should be interpreted cautiously.

## 1. Introduction

Neurological disease is currently the leading cause of disability worldwide. According to the Global Burden of Disease (GBD) 2021 study, these conditions affected approximately 3.4 billion people, representing 43.41% of the global population, and caused 11.1 million deaths annually [1]. In Europe, neurological disorders are the third leading cause of disability and premature death, after cardiovascular diseases and cancer, accounting for 13.3% of total (Disability-Adjusted Life Years) DALYs, a metric that combines years of life lost due to premature mortality and years lived with disability [2]. The population aged over 65 years bears the greatest burden of conditions such as stroke and dementia, and the overall burden is higher in women [3]. Furthermore, Parkinson’s disease shows an increasing trend in Europe, whereas stroke and neurological infections have declined in age-standardized rates, although their absolute burden remains high [3].

Malnutrition in patients with neurological disorders primarily arises through two main mechanisms: reduced dietary intake, related to conditions such as dysphagia, cognitive impairment, apraxia, depression, or functional limitations, and hyper-catabolism during acute/early stages of disease [4]. Dysphagia, most often caused by neurological conditions, markedly increases the risk of malnutrition [5]. The neurological diseases with the greatest nutritional impact include stroke, in which more than 50% of patients develop post-stroke dysphagia and around 12.5% of older patients with dysphagia become malnourished [1,3]; Alzheimer’s disease and other dementias, with malnutrition rates of 20–30% [1,3]; Parkinson’s disease, with reported malnutrition rates ranging from 0% to 24% and risk between 3% and 60% [4]; amyotrophic lateral sclerosis and Huntington’s disease, where up to 67.6% of patients are at risk of malnutrition [5]; and multiple sclerosis, in which malnutrition prevalence varies depending on disease stage [6]. Overall, neurological conditions with the greatest nutritional burden are those combining dysphagia, cognitive decline, and functional impairment. Early nutritional screening and comprehensive, multidimensional assessment of swallowing are therefore essential in this patient population [7].

Body composition in neurological diseases remains poorly characterized, largely due to the specific ways these conditions affect muscle mass and function. Reliable tools are needed to assess both muscle composition and performance, as these disorders have a significant nutritional component and patients should be evaluated and treated at different stages of the disease [8]. Furthermore, there is a lack of accurate data on the nutritional impact of neurological conditions, which limits understanding of their true burden. These diseases are also often characterized by low incidence and prevalence, as well as marked heterogeneity in onset and progression, making systematic assessment challenging [8]. Nutritional evaluation plays a key role in categorizing nutritional interventions and disease progression, detecting dysphagia early, and assessing the impact of nutrition on quality of life [8].

Muscle ultrasound has emerged as a useful, non-invasive technique for assessing both muscle quantity and quality at specific anatomical sites. It requires relatively simple equipment and, when performed by trained professionals, can be quick and feasible within the routine clinical practice. This technique allows for the evaluation of structural parameters such as muscle thickness, cross-sectional areas, and circumference, as well as qualitative aspects such as echogenicity and pennation angle [9]. As a result, muscle ultrasound provides a more comprehensive assessment of muscle health, contributing to the diagnosis of conditions such as malnutrition, according to Global Leadership Initiative on Malnutrition (GLIM) criteria [10] and sarcopenia, based on European Working Group on Sarcopenia in Older People 2 (EGWSOP2) guidelines [11]. Despite its potential, muscle ultrasound presents several limitations. Scientific evidence in neurological populations remains limited, with most studies conducted in healthy older adults rather than in patients with disease-related malnutrition. There is also considerable heterogeneity in measurement protocols, including the choice of muscle groups and uncertainty regarding whether regional measurements accurately reflect whole-body muscle mass [12]. Additionally, intra- and inter-observer variability, along with the lack of standardized measurement procedures, may affect reproducibility and comparability of results. These limitations highlight the need for further research and standardization before muscle ultrasound can be fully established as a robust tool in the nutritional and functional assessment of patients with neurological disorders [13].

Artificial intelligence (AI) offers significant advantages in nutritional assessment of muscle ultrasound by enabling objective, reproducible, and detailed analysis of muscle structure and quality. Cloud-based tools using convolutional neural networks (CNNs) with U-Net architecture allow for fully automated segmentation of the rectus femoris region of interest, achieving excellent agreement with human observers [14]. In addition, AI-driven algorithms such as Multi-Otsu histogram analysis can classify tissue composition based on echogenicity, distinguishing muscle tissue, fat infiltration, and fibrotic changes, thereby providing a more precise evaluation of muscle quality. These systems can also extract quantitative radiomic biomarkers, including anatomical, textural, and fractal features, expanding the diagnostic information beyond conventional measures. Overall, the integration of AI into muscle ultrasound reduces inter-observer variability, enhances diagnostic accuracy, and facilitates early detection of malnutrition and sarcopenia, as well as objective monitoring of nutritional interventions in clinical practice [15].

Muscle ultrasound is a promising technique for assessing muscle impairment and for the diagnosis of malnutrition and sarcopenia. However, it still presents several limitations, including the lack of well-defined measurement criteria, uncertainty about the most appropriate muscle groups to evaluate, and limited scientific evidence supporting its use. Further research is needed to validate this technique in a variety of neurological disorders. In addition, the application of AI to ultrasound imaging may enable the identification of novel variables related to muscle quality, improving diagnostic accuracy and enhancing prognostic assessment.

The purpose of this study was to validate whether integrating AI into muscle ultrasound can identify novel muscle quality in ways that overcome current methodological limitations. For this reason, a study is designed to evaluate the performance of an AI-based tool for quantitative and qualitative analysis of muscle ultrasound images. The protocol aims to compare analysis of ultrasound-derived profiles across a spectrum of neurological disorders to determine whether AI-extracted features discriminate conditions with direct neuromuscular involvement from those without. Finally, the study aim is also to assess the long-term prognostic value of novel AI-derived muscle quality indices, for predicting clinically relevant outcomes such as mortality and the presence and severity of dysphagia, thereby evaluating their potential integration into routine diagnostic and prognostic workflows.

## 2. Materials and Methods

### 2.1. Design

This was a prospective, open-label observational study designed to evaluate the nutritional status and clinical evolution of patients based on standard nutritional interventions (adapted dietary recommendations and/or oral supplementation).

The study specifically investigated the relationship between ultrasound-derived variables obtained through artificial intelligence (AI) and classical measures of body composition. Ultrasound images of patients with neurological diseases at risk of malnutrition were collected by a single investigator using the same device. These images were analyzed both by conventional observer assessment and through an on-line AI-powered platform through conventional observer and via an online AI-powered platform for body-composition assessment (https://dawako.es, accessed between November and December of 2024). Importantly, no personal or health-related information was uploaded to the platform, only the ultrasound images themselves.

After informed consent and patient inclusion, a comprehensive anamnesis was performed, including demographic data, medical history, disease progression, and nutritional background. Each patient underwent anthropometry, bioimpedance analysis, handgrip strength testing, and muscle (quadriceps rectus femoris, QRF) ultrasound evaluation. In addition, in patients identified as being at risk of dysphagia, the Volume-Viscosity Swallow Test (MECV_V) was performed as part of the clinical assessment. Medical and nutritional treatments prescribed were recorded. The primary outcomes of this study were clearly defined as mortality and the presence of dysphagia (assessed via the Volume-Viscosity Swallow Test), alongside secondary metrics like hospital admissions and emergency department visits. Patient data were collected two years after study initiation to assess clinical outcomes, focusing on mortality, hospitalization rates, and emergency department visits.

The collected data allowed for a descriptive statistical analysis of prevalence, nutritional status, and muscle condition (quantity and quality), comparing classical body-composition assessment techniques with AI-based ultrasound analysis. Functional status was assessed through handgrip dynamometry. Inferential univariate and multivariate analyses were conducted to evaluate the impact of nutritional support on clinical outcomes, body-composition variables, and their potential modification of patient prognosis.

The Ethics Committee for Research with Medicines (CEIm) of the Valladolid Health Areas (approval code: PI 22-2910 on 13 October 2022 for nutritional assessment and PI-24-622-C for AI evaluation of the images, on 20 November 2024) formally authorized the conduct of this study. All procedures were carried out in strict accordance with the ethical standards of the institutional research committee and adhered to the principles outlined in the Declaration of Helsinki.

### 2.2. Patients

Patients were recruited from the Clinical Nutrition Outpatient Clinic at the Hospital Clínico Universitario de Valladolid between October 2022 and December 2024. All individuals attended the clinic following a referral issued by the Neurology Department, in accordance with routine clinical practice. Consequently, the initial suspicion of malnutrition was based on the clinical judgement of the referring neurologist, and no standardized malnutrition screening tool was applied prior to the GLIM evaluation.

A consecutive sampling strategy was applied in the clinic. Eligible participants were outpatients aged >18 years with high nutritional risk and a confirmed diagnosis of a neurological disorder; patients with decompensated liver disease, chronic kidney disease stage IV or higher, or without signed informed consent were excluded. No formal sample size calculation was performed a priori because of the heterogeneity of the target population and the limited prior evidence regarding the novel technique under study (AI-assisted muscle ultrasonography).

### 2.3. Variables

#### 2.3.1. Anthropometric Measures

The anthropometric variables assessed included the following: current body weight (kg), defined as the weight measured at the time of clinical evaluation; usual body weight (kg), referring to the patient’s typical weight during the months preceding the pathological condition associated with malnutrition, obtained through clinical interviews and review of medical records when available; height (m); body mass index (BMI, calculated as current weight/height^2^, kg/m^2^); arm circumference (cm); calf circumference (cm); and percentage of body weight loss, calculated as (usual weight − current weight)/usual weight × 100.

#### 2.3.2. Electrical Bioimpedanciometry

Bioelectrical impedance analysis was performed using a BIA 101 Anniversary device (EFG, Akern, Pisa, Italy). Measurements were obtained between 8:00 and 10:00 AM after an overnight fast, following a 15-min rest in the supine position. The raw electrical parameters recorded included reactance (ohm), resistance (ohm), and phase angle (°). The appendicular skeletal muscle index (ASMI), employed to diagnose low muscle mass and malnutrition, was calculated using Sergi’s formula [16].

#### 2.3.3. AI-Based Muscular Ultrasonography

Ultrasonographic assessment of the quadriceps rectus femoris (QRF) muscle was carried out on the dominant lower limb using a 10 MHz multifrequency 7L4P linear probe in musculoskeletal mode from Mindray Z60 equip (Mindray, Madrid, Spain). Measurements were obtained with the patient in a supine position, placing the probe perpendicular to the muscle along the transverse axis of the dominant leg, at the lower third of the distance between the iliac crest and the superior border of the patella [9]. All examinations were performed by trained personnel following a standardized protocol. The imaging parameters were consistently applied across participants to ensure reproducibility: frequency 10 MHz, depth 4.5–4.7 cm, gain 43–45, frame rate 23 frames per second, and dynamic range 155 to minimize inter-operator variability and ensure reproducibility. Operators received specific training in muscle imaging techniques, including probe placement, image optimization, and minimizing tissue compression to prevent distortion. During acquisition, only minimal pressure was applied to the limb, sufficient to obtain a clear image without altering the subcutaneous tissue or underlying muscle architecture. Images were archived in JPEG format. While JPEG compression is inherently lossy, careful handling ensured that diagnostic information remained uncompromised. To maintain quality control and blinding, the image processing and AI-based analysis were performed by a different individual than the investigator who initially acquired the ultrasound images.

Ultrasonographic images were processed using an AI-based imaging platform (PIIXMEDTM; DAWAKO MedTech; Valencia, Spain). PIIXMEDTM is a cloud-based diagnostic support system designed for automatic segmentation and analysis of medical ultrasound images. It employs a convolutional neural network (CNN) with a U-Net architecture, originally developed at the University of Freiburg [17], optimized to achieve accurate segmentation with relatively few training images. The system enables 2D feature extraction from conventional B-mode ultrasound and calculates single feature values within a defined region of interest (ROI). Radiomics-based algorithms, implemented through an open-source Python package (version 13.2) [18], allow for quantitative extraction of anatomical measurements, echogenicity, texture, and fractal dimension. These features serve as surrogate biomarkers of muscle mass and quality. Biomarkers were derived by analyzing ROI morphology, echogenicity-based muscle quality and texture indices, with further details presented later.

Segmentation performance has been validated previously by García-Herreros et al., demonstrating high intra-class correlation coefficients compared with human observers: 0.912 for subcutaneous fat, 0.96 for muscle thickness, and 0.99 for muscle area [14]. Muscle mass parameters included rectus femoris muscle area (RFMA, cm2) and rectus femoris muscle thickness (RFMT, cm), representing cross-sectional area and thickness of the muscle belly in transverse section. Subcutaneous fat thickness (SFT) was measured in longitudinal section to quantify adipose tissue depth.

**Figure 1:**
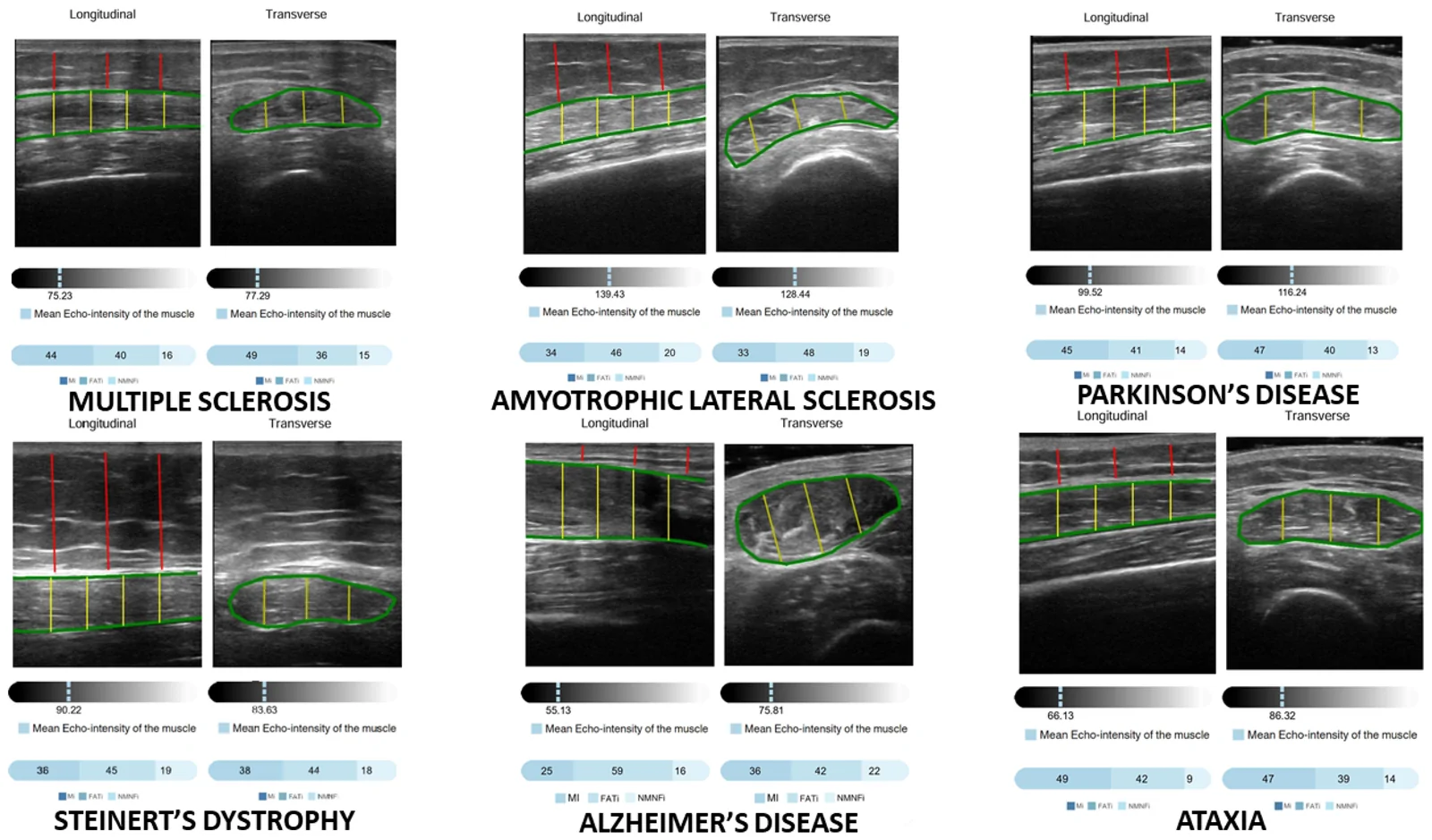
Different rectus femoris ultrasound images analyzed by Artificial Intelligence tool (PIIXMEDTM). Mi: Low Echogenicity Index (Muscle Index); FATi: Medium Echogenicity Index (Fat Index); NMNFi: High Echogenicity Index (No Muscle No Fat index). Red line: adipose tissue, green line: muscle fascia; yellow line: muscle thickness.

Muscle quality was assessed by pennation angle (degrees) in longitudinal section, defined as the angle between muscle fibers and the lower aponeurosis; larger angles indicate greater potential for force generation. Additional quality indices were obtained using a multi-thresholding algorithm based on histogram echogenicity and gray-level intensity. Specifically, the Multi-Otsu algorithm, an extension of the traditional Otsu method, partitions pixels into three or more classes rather than two. Applied to muscle ultrasound, this approach classifies tissue regions by echogenicity: low (muscle tissue, Mi), medium (fat tissue, FATi), and high (non-muscle/non-fat, NMNFi). Thresholds are computed to minimize intra-class variance and maximize inter-class variance, yielding refined segmentation across intensity levels [19]. In practice, the algorithm generates two threshold values, visualized as vertical violet lines on the histogram, and reports indices as percentages of the ROI. The AI tool outputs ROI segmentation, muscle mass values, and muscle quality indices expressed as percentages of echogenicity classes. Although capable of analyzing both longitudinal and transverse indices, this study focused on transverse measures, as they are more consistently supported in the literature, easier to standardize, and associated with lower inter-observer variability (Figure 1).

#### 2.3.4. Muscle Strength

Handgrip strength, measured with a JAMAR Hydraulic Hand Dynamometer, (JAMAR^TM^, Basel, Switzerland), was used to assess muscle function. The test was conducted with patients seated, maintaining their dominant arm at a right angle to the forearm while executing the handgrip.

#### 2.3.5. Intake Assessment

Dietary intake was evaluated using a prospective 2-day food record, and subsequently analyzed with Dietsource^TM^ software (Nestlé, Geneva, Switzerland). This analysis yielded total daily energy intake and macronutrient consumption for carbohydrates, fats, and proteins, together with the proportional contribution of each macronutrient to overall energy intake. Intake was examined both in absolute terms and adjusted for body weight, including energy per kilogram and protein per kilogram. In addition, adherence to individual energy and protein requirements was quantified by calculating the percentage of estimated need achieved for each patient.

Energy requirements were estimated by calculating basal expenditure with the revised Harris–Benedict equation and applying an activity/stress factor of 1.5, reflecting either preserved mobility or a moderate-to-severe disease-related stress load in patients with paradoxical hyper-catabolism [8,20]. Protein needs were determined on a weight-adjusted basis, following ESPEN recommendations for neurological patients, which generally support a target of approximately 1.5 g/kg/day in this population [8].

#### 2.3.6. Biochemical Parameters

Biochemical assessments were performed using a Cobas c711 automated analyzer (Roche Diagnostics, Basel, Switzerland). The panel of nutritional and inflammatory markers included albumin, expressed in gram per deciliter (g/dL); C-reactive protein (CRP) reported in milligrams per deciliter (mg/dL); prealbumin, also measured in milligrams per deciliter (mg/dL); and the CRP-to-prealbumin ratio and CRP-to-albumin ratio, which offer an integrated indication of the interplay between inflammatory activity and nutritional status.

#### 2.3.7. Malnutrion and Sarcopenia Diagnosis

- Malnutrition Diagnosis: The diagnosis of malnutrition was established according to the Global Leadership Initiative on Malnutrition (GLIM) criteria, which require the presence of at least one phenotypic criterion together with one etiologic criterion. In our cohort, all patients inherently fulfilled the etiologic component due to the underlying neurological disease burden associated with their primary condition. Nevertheless, malnutrition prevalence was also assessed considering the etiologic criteria of intake below 75% and CRP above 3 mg/L. The classification of malnutrition severity was therefore based on the phenotypic indicators specified by GLIM, namely unintentional weight loss and reduced BMI [10].
- Sarcopenia Diagnosis: Sarcopenia was diagnosed according to the European Working Group on Sarcopenia in Older People (EWGSOP2) criteria [11]. Diagnosis required both reduced handgrip strength, defined as <27 kg in men and <16 kg in women, and low muscle mass, defined as an appendicular skeletal muscle index (ASMI) <7 kg/m^2^ in men and <5.5 kg/m^2^ in women, as determined by bioelectrical impedance analysis (BIA). Patients presenting with reduced handgrip strength but preserved muscle mass were classified as having probable sarcopenia, also named dynapenia.
- Sarcopenic Obesity: Sarcopenic obesity was diagnosed according to the joint European Society of Clinical Nutrition and Metabolism (ESPEN) and European Association for the Study of Obesity (EASO) consensus criteria [21]. The diagnosis required the coexistence of low muscle mass, defined as an appendicular skeletal muscle index (ASMI) <7.0 kg/m^2^ in men and <5.5 kg/m^2^ in women, together with reduced muscle strength, assessed through handgrip strength (<27 kg in men and <16 kg in women). Excess adiposity was determined using body fat percentage obtained by bioelectrical impedance analysis (BIA), applying sex-specific cut-offs (≥25% in men and ≥35% in women). Patients presenting low muscle mass and dynapenia in the presence of excess fat mass were classified as having sarcopenic obesity [21]

#### 2.3.8. Clinical Variables

Among the neurological diseases that have been investigated are motor neuron disease, multiple sclerosis, Parkinson’s disease, and Steinert’s myotonic dystrophy, as well as other conditions such as stroke, dementia, and cerebral palsy. Due to differences in prevalence and disease course, these conditions were analyzed separately, but they were also classified into two groups: those with direct neuromuscular involvement (Parkinson’s disease, motor neuron disease, multiple sclerosis, and Steinert’s myotonic dystrophy) and those without direct neuromuscular involvement (dementia, cerebral palsy, stroke) and other neurological disorders (ataxia, polyneuropathy, metabolic encephalopathy, functional neurological disorder).

#### 2.3.9. Swallowing Assessment

Assessment of swallowing function was performed using the Volume-Viscosity Swallow Test (MECV_V) in patients identified as being at risk of dysphagia, except in cases of motor neuron disease, for which the procedure is performed at the time of diagnosis in accordance with the clinical protocol. This clinical bedside test evaluates swallowing safety and efficacy by administering boluses of different volumes (5, 10, and 20 mL) and viscosities (thin liquid, nectar-like, and pudding-like). Clinical signs such as cough, voice changes, and oxygen desaturation were monitored to detect impaired swallowing safety, while oral and pharyngeal residue were assessed to determine swallowing efficacy. The test was conducted by trained clinicians following standardized procedures to ensure reproducibility and patient safety [22].

Based on these findings, patients were classified into three categories: those with impaired safety, those with impaired efficacy, and those with impairment in both domains. In cases where further evaluation was required, a fiberoptic endoscopic evaluation of swallowing (FEES) was performed, and the results were recorded as either positive or negative. All assessments were conducted by trained clinicians following standardized procedures to ensure reproducibility and patient safety.

#### 2.3.10. Outcomes

The primary outcome variables evaluated were mortality, hospital admissions, and emergency department visits. In addition, dysphagia, classified according to impaired safety, impaired efficacy, or impairment in both domains, was considered as prognostic variable. Follow-up was conducted for two years after the baseline consultation and assessments to record mortality, hospital admissions, and emergency department visits. Dysphagia was evaluated at the baseline consultation.

### 2.4. Statistical Analysis

The information was stored in a licensed database using the statistical software SPSS version 23.0 (SPSS Inc., Chicago, IL, USA), under the official license of the University of Valladolid. Normality of continuous variables was assessed using the Kolmogorov–Smirnov test. Continuous variables with normal distribution were expressed as mean (standard deviation), whereas those with no normal distribution were expressed as median (25th–75th percentile). Categorical variables were presented as absolute numbers and percentages. Parametric variables were analyzed using paired and unpaired Student’s *t*-tests, while non-parametric variables were assessed with Friedman, Wilcoxon, Kruskal–Wallis, and Mann–Whitney U tests. For comparisons involving more than two groups, ANOVA with Bonferroni post hoc correction was applied. Categorical variables were analyzed using the chi-square test, with Fisher’s or Yates’ corrections when appropriate.

Additionally, a multivariate analysis was also conducted to assess the association between changes in the measured variables and patient prognosis. In the multivariate analysis used to identify predictors of death, the model adjusted for several key variables, including age (specifically >65 years), sex, neuromuscular involvement, and various AI-derived echogenicity indices such as FATi, Mi and NMNFi. Echogenicity values were expressed as dichotomous variables; classifying patients as above or below the median. The median values for these parameters were: FATi (41.20 (37.26–45.14)%); Mi (43.12 (38.74–49.45)%) and NMNFi (14.82 (11.95–17.53)%). The model calibration was evaluated using the Hosmer–Lemeshow goodness of fit test to assess the adequacy of the multivariate logistic regression model.

## 3. Results

### 3.1. Sample Description

A total of 117 patients were included in the analysis. Among them, 61 patients (52.1%) were women, with a mean age of 63.01 (16.14) years.

**Figure 2:**
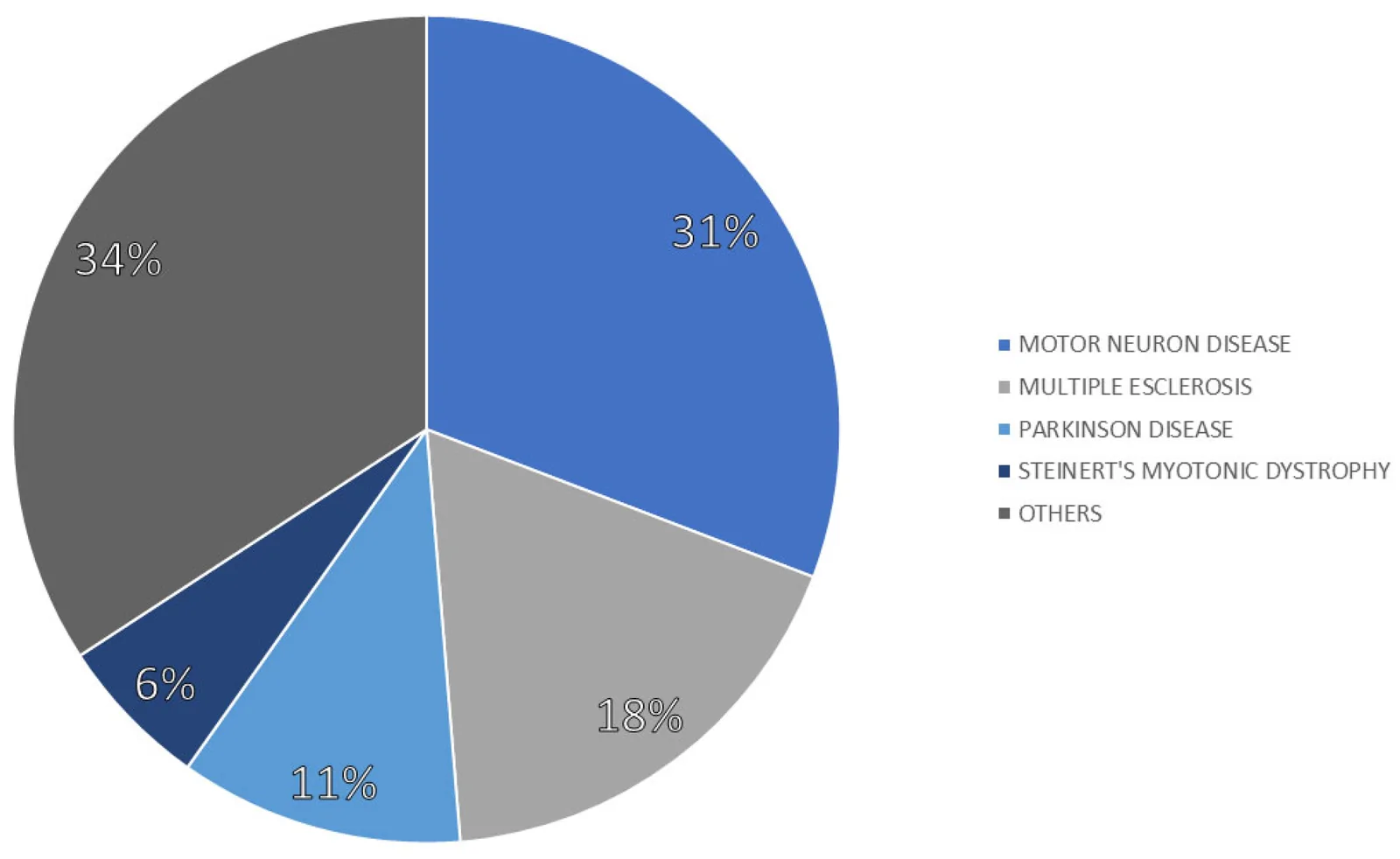
Distribution of neurologic pathologies.

The patients presented with a range of neurological conditions, as illustrated in Figure 2. Of the sample, 77 patients (65.8%) had a condition with direct neuromuscular involvement, while 40 patients (34.2%) had a condition without direct neuromuscular involvement.

**Figure 3:**
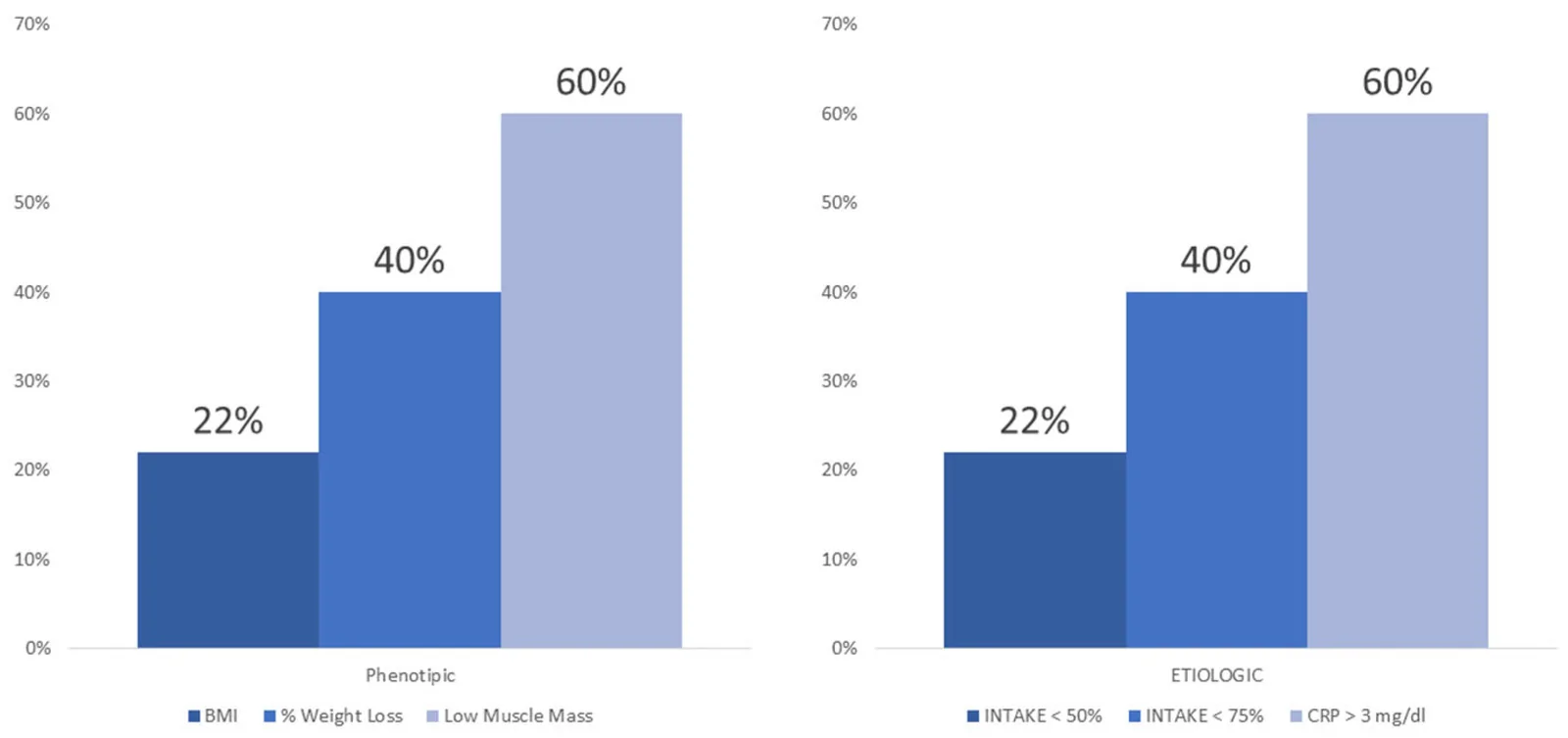
Percentage of patients with each of the GLIM components. BMI: Body Mass Index; CRP: C-Reactive Protein.

According to the GLIM criteria, 73 patients (62.4%) were identified as malnourished, with 30 patients (25.6% of the total cohort) presenting severe malnutrition. All patients were considered as meeting the etiologic criteria due to the underlying neurologic disease burden. Nevertheless, when applying the full GLIM framework, we observed a distinct distribution of phenotypic and etiologic components (Figure 3). The phenotypic panel illustrates the relative contribution of reduced BMI, weight loss, and diminished muscle mass, highlighting how each parameter shapes the clinical expression of malnutrition. The etiologic panel depicts the proportion of patients with insufficient intake and evidence of inflammation, offering a complementary view of the underlying nutritional risk. Together, these panels provide a comparative visualization of how GLIM criteria manifest across the population. If we only consider etiologic criterion as intake below 75% or CRP above 3 mg/dL, 24.8% of patients had malnutrition.

Furthermore, 70 patients (59.8%) exhibited dynapenia, among whom 41 (35% of the total) fulfilled the criteria for sarcopenia. A total of 22 patients (18.8%) had sarcopenic obesity (SO).

Significant sex differences were observed in absolute parameters, including BMI, arm circumference, bioimpedance resistance and reactance, handgrip strength, and ultrasound dimensions (RFMA, RFMT, SFT) (Table 1). In contrast, muscle quality measures expressed as percentages of different echogenicity areas from ROI did not show significant differences between sexes.

There was an intake below the recommended requirements, more marked in protein intake and in men compared with women (Table 1). Regarding the route of nutritional support, 14 patients (13.9%) were receiving enteral nutrition through feeding tubes (nasogastric tube or gastrostomy). When comparing anthropometric parameters between individuals with tube feeding and those without, no significant differences were observed in the comprehensive nutritional assessment except for calf circumferences, which was lower in patients with enteral access (30.25 (9.32) cm) compared with those without tube feeding (33.41 (4.13) cm); *p* = 0.04. There were no differences in biochemical parameters between sexes (Table 1).

Patients with sarcopenic obesity (SO) had older age (SO: 73.77 (9.95) years vs. Non SO: 60.52 (16.31) years; *p* < 0.01) and had worse muscle quality parameters (Mi: SO: 39.80 (8.04%) vs. Non SO: 45.15 (8.87%); *p* = 0.01); (FATi: SO: 43.21 (5.42%); vs. Non SO: 40.45 (5.51%); *p* = 0.03) and (NMNFATi: SO: 16.99 (4.05%); Non SO: 14.48 (5.23%); *p* = 0.04).

### 3.2. Differences in Ultrasonographic Parameters Between Different Neurological Entities

There were no differences in sex between patients with direct neuromuscular involvement and those without. AI-powered ultrasound parameters were compared between patients with and without neurodegenerative disease. There were no differences in muscle mass parameters, but patients with neuromuscular involvement had lower values of percentage of low-echogenicity areas in ROI (Mi), assumed as muscle, and higher values of medium-echogenicity areas, assumed as fat (Fati, and high-echogenicity areas, assumed as no muscle/no fat (NMNFi) (Table 2).

Among patients with direct neuromuscular involvement, the four diagnostic groups (motor neuron disease, multiple sclerosis, Parkinson’s disease, and Steinert’s myotonic dystrophy) showed significant differences in age, weight loss, bioelectrical impedance parameters, body composition, and handgrip strength. Patients with multiple sclerosis exhibited the highest phase angle and handgrip strength, whereas those with Parkinson’s disease were the oldest and experienced the greatest weight loss. Patients with multiple sclerosis had more energy protein consumption and better adjustment to requirements without differences in biochemical parameters. Complete results are presented in Table 3.

### 3.3. Relationship Between Classic Variables with AI-Derived Muscle Ultrasound Variables

Nutritional and functional status in patients with neuromuscular involvement are closely linked to specific muscle ultrasound parameters. Quantitative indices, such as the area and thickness of the rectus femoris, demonstrate significant positive correlations with anthropometric measures and functional biomarkers like phase angle and handgrip strength, while being inversely related to bioimpedance resistance. Regarding muscle quality, the presence of low-echogenicity tissue is positively associated with better functional performance. These architectural and qualitative variations, alongside their clinical correlations, are further detailed in Table 4.

FATi values above the median were independently associated with the presence of neurodegenerative disease (OR = 3.02; 95%CI: 1.26–7.26; *p* < 0.01), after adjustment for sex, age, and BMI.

### 3.4. Differences in Ultrasonographic Parameters Related to Outcomes

In this cohort, the prevalence of mortality was 26 patients (22.2%), while 39 patients (33.3%) required hospital admission. Emergency department utilization was also frequent, with 69 patients (59%) presenting at least once and a median of 1 (0–3) visits per patient.

In the comparison between deceased patients and survivors, those who died were generally older and showed greater weight loss, reduced arm circumference, and lower appendicular muscle mass. Ultrasound assessment of the rectus femoris revealed a lower proportion of muscle tissue and higher fat infiltration in the deceased group. Handgrip strength was also markedly diminished. In contrast, no relevant differences were observed in sex distribution, BMI, calf circumference, bioimpedance parameters, or other ultrasound indices. These findings are summarized in Table 5 Nutritional assessment did not differ in relation to emergency department visits or hospital admissions.

The Volume-Viscosity Swallow Test was performed in 99 patients (84.6% of the cohort). Safety alterations were observed in 39 patients (39.4%), efficacy alterations in 52 patients (44.4%), and both alterations in 23 patients (19.7%). FEES was performed in 72 patients (61.5%); 33 had positive findings, corresponding to 45.8% of those tested and 28.2% of the total cohort.

Patients with dysphagia in the Volume-Viscosity Swallow Test had lower values of BMI (dysphagia: 23.29 (5.44) kg/m^2^; no dysphagia: 25.97 (5.29) kg/m^2^; *p* = 0.03) and calf circumference (dysphagia: 32.12 (5.88) cm; no dysphagia: 34.33 (3.58) cm; *p* = 0.03). There were no differences in muscle mass parameters of muscular ultrasound neither in RFMA (dysphagia: 3.39 cm^2^; no dysphagia: 3.44 cm^2^; *p* = 0.85); nor RFMT (dysphagia: 1.13 (0.34) cm; no dysphagia: 1.11 (0.31) cm; *p* = 0.76), and in terms of muscle quality there was no difference in Mi (dysphagia: 45.02 (9.96)%; no dysphagia: 41.35 (7.56)%; *p* = 0.05) but FATi was slightly higher in patients without dysphagia (dysphagia: 40.47 (5.70%); no dysphagia: 42.71 (5.25%); *p* < 0.05).

The confirmation of dysphagia was performed by swallow videoendoscopy and 33 patients (28.2%) had confirmed dysphagia. Patients with sarcopenic obesity had more dysphagia (10 patients, 45.5%) than those without (23 patients, 24.2%); *p* = 0.03.

### 3.5. Relation Between New Ultrasonographic Parameters and Outcomes

**Figure 4:**
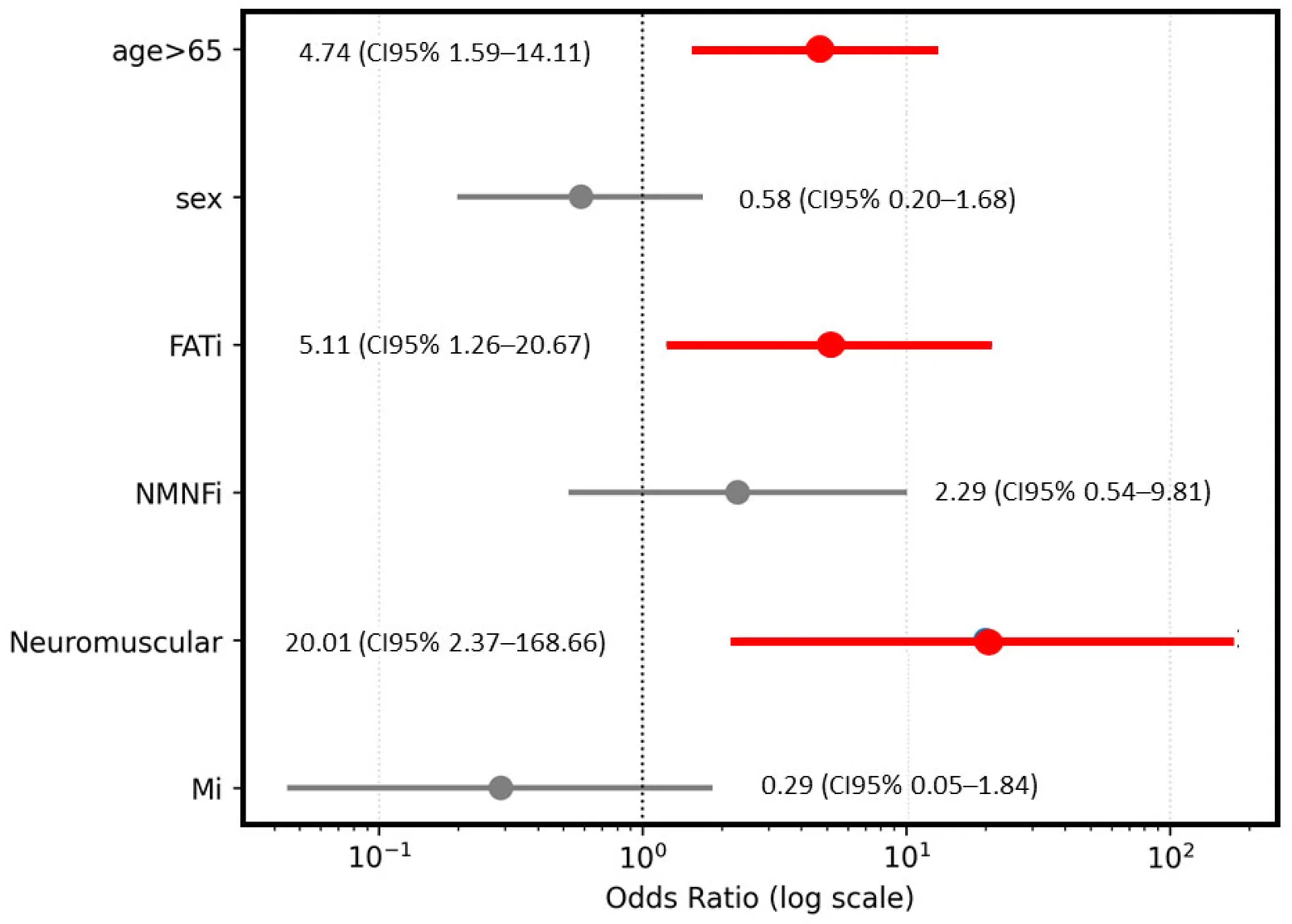
Forest plot for death risk showing odds ratios (OR) and 95% confidence intervals (CI 95%) for predictors included in the logistic regression model. Variables were coded as follows: age > 65 years (1 = yes, 0 = no), sex (1 = male, 0 = female), FATi (medium echogenicity), NMNFi (high echogenicity) (1 = value above the median (FATi: 41.20%; NMNFi: 14.82%), 0 = below), Mi (low echogenicity) (1 = value below the median (Mi: 43.12%), 0 = above), and neuromuscular involvement (1 = present, 0 = absent). The plot uses a logarithmic scale for OR, with a vertical reference line at OR = 1 indicating no effect. Red markers represent statistically significant associations (*p* < 0.05).

In the multivariate analysis, FATi (above median), age > 65 years, and neuromuscular involvement increased risk of death, while sex, NMNFi, and Mi did not show relation. Figure 4 displays the odds ratios (OR) and 95% confidence intervals (CI 95%) for all predictors on a logarithmic scale. The Hosmer–Lemeshow test showed an adequate fit with a chi-square value of 4.85; *p* = 0.77.

**Figure 5:**
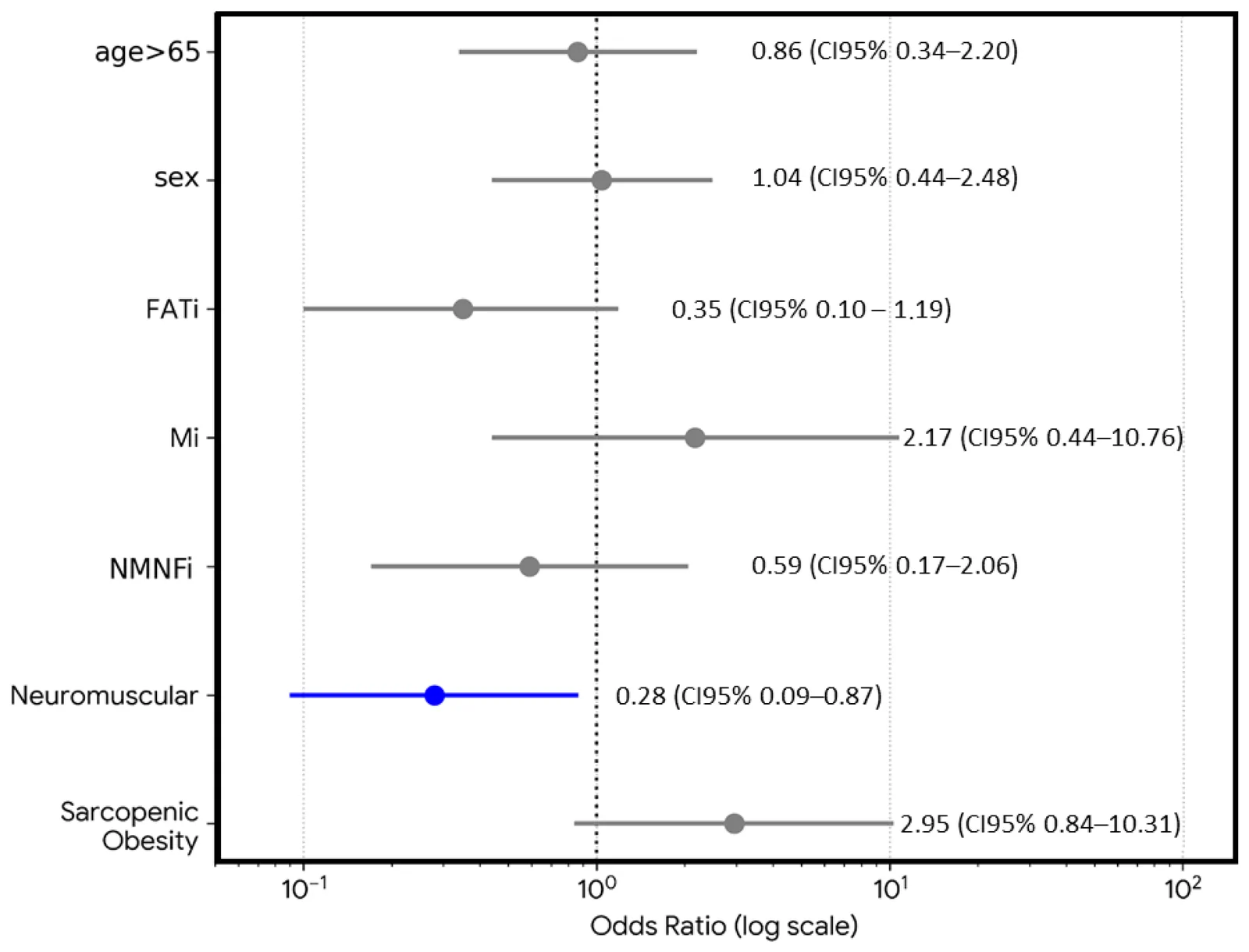
Forest plot for dysphagia risk (Volume-Viscosity Swallow Test) showing odds ratios (OR) and 95% confidence intervals (CI 95%) for predictors included in the logistic regression model. Variables were coded as follows: age > 65 years (1 = yes, 0 = no), sex (1 = male, 0 = female), FATi (medium echogenicity), NMNFi (high echogenicity) (1 = value above the median (FATi: 41.20%; NMNFi: 14.82%), 0 = below), Mi (low echogenicity) (1 = value below the median (Mi: 43.12%), 0 = above), and neuromuscular involvement (1 = present, 0 = absent). The plot uses a logarithmic scale for OR, with a vertical reference line at OR = 1 indicating no effect. Blue markers represent statistically significant associations (*p* < 0.05).

The analysis of dysphagia (Volume-Viscosity Swallow Test) in relation to the same risk factors showed an inverse association with neuromuscular involvement, probably reflecting referral or selection patterns rather than a protective biological effect, after controlling for sex, NMNFi, FATi, Mi, and the presence of sarcopenic obesity (Figure 5). The Hosmer–Lemeshow test showed an adequate fit with a chi-square value of 3.68; *p* = 0.88.

**Figure 6:**
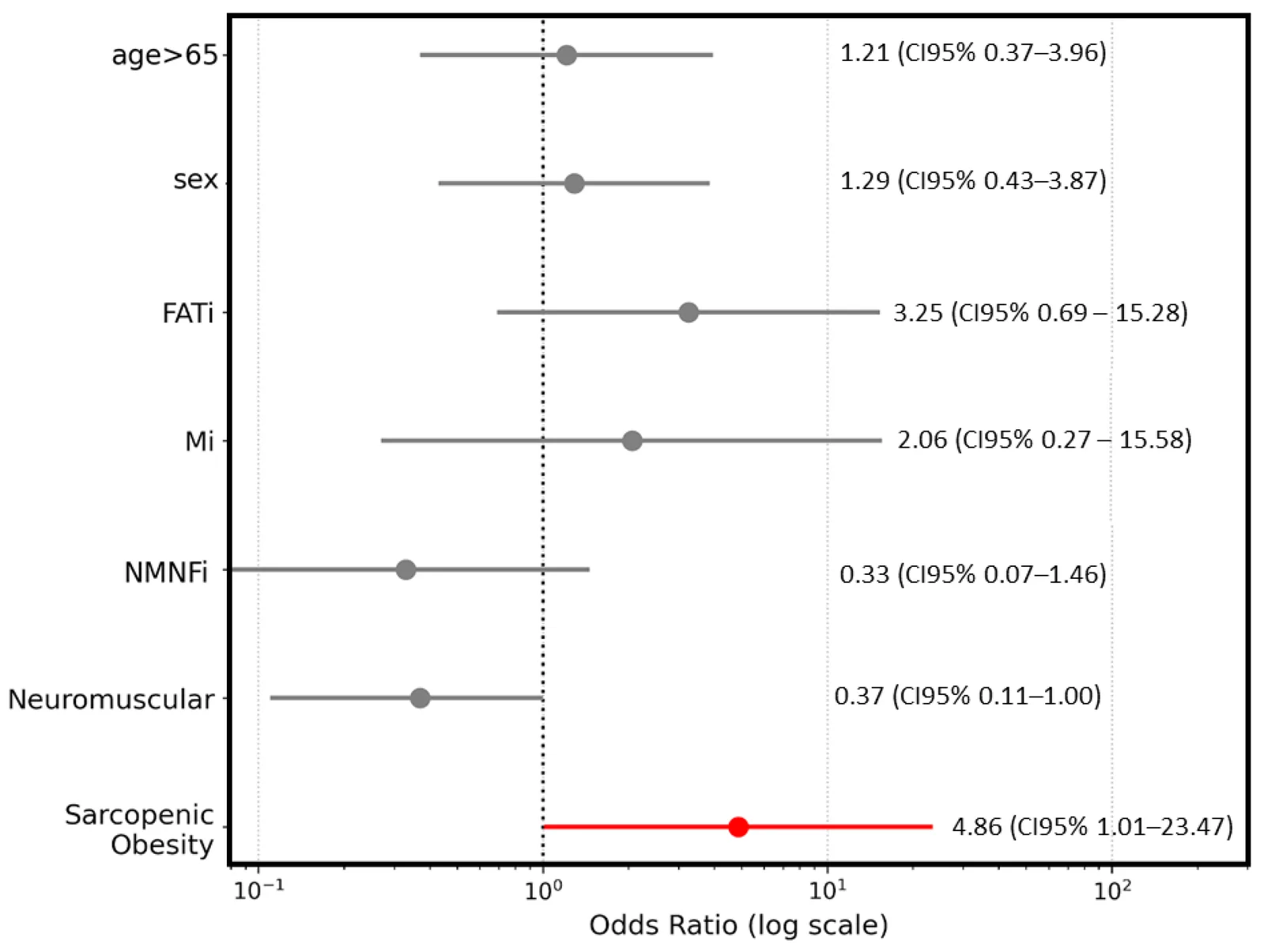
Forest plot for dysphagia (Swallow Endoscopy) showing odds ratios (OR) and 95% confidence intervals (CI 95%) for predictors included in the logistic regression model. Variables were coded as follows: age > 65 years (1 = yes, 0 = no), sex (1 = male, 0 = female), FATi (medium echogenicity), NMNFi (high echogenicity) (1 = value above the median (FATi: 41.20%; NMNFi: 14.82%), 0 = below), Mi (low echogenicity) (1 = value below the median (Mi: 43.12%), 0 = above), and neuromuscular involvement (1 = present, 0 = absent). The plot uses a logarithmic scale for OR, with a vertical reference line at OR = 1 indicating no effect. Red markers represent statistically significant associations (*p* < 0.05).

The multivariate analysis of dysphagia, evaluated directly via endoscopy, in relation to the same risk factors showed the presence of sarcopenic obesity was a significant risk factor for dysphagia (OR = 4.86; CI 95%: 1.01–23.47; *p* = 0.03). This analysis was controlled for sex, quality variables of AI-based muscle ultrasound, and neuromuscular involvement (Figure 6). The Hosmer–Lemeshow test showed an adequate fit with a chi-square value of 15.43; *p* = 0.51.

## 4. Discussion

In this study, AI-assisted muscle ultrasound revealed muscle composition patterns that were not captured by conventional muscle mass metrics of ultrasonography in adults with neurological disorders. Individuals with neuromuscular involvement showed a distinctly lower proportion of low-echogenicity muscle areas and a greater presence of medium-echogenicity regions, reflecting a shift toward increased intramuscular fat infiltration. Notably, a higher burden of these medium-echogenicity areas emerged as an independent marker of poorer prognosis, even after accounting for demographic and clinical factors.

There were no significant differences in age, BMI, or weight loss between patients with diseases affecting the neuromuscular system and those with cerebral conditions (stroke, ataxia, dementia, or cerebral palsy). Most patients were referred to the Clinical Nutrition Service due to malnutrition risk, which may explain this similarity. This contrasts with published literature, where neurodegenerative diseases involving motor neurons or muscle show the most pronounced and prognostic body-composition changes [23,24,25], Parkinson’s disease presents moderate fat-predominant loss [26], multiple sclerosis is uniquely worsened by obesity [27], and stroke/ataxia exhibit secondary nutrition decline driven by disability rather than intrinsic metabolic dysfunction [25]. Our sample likely differs because all patients were evaluated in a Clinical Nutrition setting with established malnutrition risk.

Regarding muscle mass (estimated ASMI, RFMT, RFMA), no significant differences were observed. However, muscle quality (low Mi and high FATi) was poorer in patients with neuromuscular involvement, and this was associated with lower handgrip strength. Neuromuscular disease such as Amyotrophic Lateral Sclerosis (ALS) and Steinert’s Disease (SD) primarily show early structural muscle deterioration detectable on imaging before functional decline [28,29]. In contrast, brain-origin conditions like stroke display disproportionate functional weakness relative to structural changes due to central neural impairment. SD exhibits the greatest myosteatosis, ALS the fastest deterioration in muscle quality, and stroke uniquely causes bilateral, rapid, and regionally heterogeneous muscle quality loss. Muscle quality begins to deteriorate within the first 10 days after stroke onset, with significant echo intensity increases in the paretic limb; in chronic stroke, intramuscular fat is independently associated with muscle strength [30,31].

Among patients with direct neuromuscular involvement Parkinson’s disease are generally the oldest and experience the most significant weight loss, while those with multiple sclerosis tend to be the youngest. Regarding muscle quality and strength, multiple sclerosis patients exhibit the highest handgrip strength, the highest phase angle and the highest Mi. In contrast, patients with Steinert’s Myotonic Dystrophy show the lowest handgrip strength and the lowest phase angle, but they have the highest medium echogenicity index (FATi) and the greatest subcutaneous fat thickness. Motorneuron disease and Parkinson’s patients fall into intermediate ranges for many of these ultrasound and nutritional metrics, probably related to referral at an earlier disease stage. Muscle quality deteriorates earliest and most intensely in ALS, where echogenicity rises rapidly and predicts functional decline better than muscle thickness or clinical scales [28]. SD shows a distinct pattern of severe myosteatosis, with fat fraction strongly linked to disease severity and reduced functional capacity [32]. In multiple sclerosis, muscle quality deficits are subtle but detectable, reflecting disuse and corticospinal dysfunction rather than primary myopathy [33]. In Parkinson’s disease, structural muscle quality is relatively preserved, yet contractile function and power are markedly impaired due to central motor dysfunction [34].

Deceased patients were significantly older and showed lower muscle mass, reduced handgrip strength, and poorer muscle quality parameters on AI-assisted muscle ultrasound. In the multivariate analysis, higher FATi increased the risk of death after adjustment for age, neuromuscular involvement, and sex. This indicates that poor muscle quality is associated with mortality in patients with neurological disease. In ALS, the relationship between muscle quality and prognosis is particularly direct: reduced muscle thickness and low CT muscle density provide prognostic information superior to standard clinical scales [35,36]. On the other hand, in patients with ALS, higher echogenicity in muscle ultrasound is seen in patients who died [13]. In stroke and Parkinson’s disease, impaired muscle quality predicts the downstream cascade of disability, falls, and frailty that ultimately contributes to mortality [37,38]. In SD, muscle quality is incorporated into broader composite prognostic models, although it has not yet been identified as an independent mortality predictor [39]. Taking together, these patterns support our finding of elevated intramuscular fat on ultrasound, as conditions with neuromuscular or central motor pathway involvement consistently show early and clinically meaningful deterioration in muscle quality.

Dysphagia is essential as this symptom represents the critical intersection between malnutrition and muscle impairment in patients with neurological disorders, where oropharyngeal dysphagia directly impacts malnutrition risk as an independent predictor. Patients who showed dysphagia on the Volume-Viscosity Swallow Test had lower BMI and smaller calf circumference, indicating a more advanced nutritional decline. However, muscle mass measured by muscle ultrasound did not differ between patients with dysphagia and non-dysphagia patients. Regarding muscle quality, Mi values were similar, but FATi was paradoxically higher in patients without dysphagia, suggesting that intramuscular fat infiltration was not the primary driver of swallowing impairment in this cohort. Patients without dysphagia showed higher BMI and higher FATi values, a pattern that may be partly explained by the presence of sarcopenic obesity, in which excess adiposity coexists with impaired muscle quality. Additionally, neuromuscular disease emerged as an independent predictor of increased FATi, while simultaneously acting as a protective factor against dysphagia due to earlier referral to nutrition services, often before swallowing impairment develops. In contrast, intramuscular fat infiltration can be elevated even when muscle thickness is preserved, consistent with geriatric evidence that echo-derived adipose infiltration predicts dysphagia more strongly than muscle size. Therefore, the underlying disease type may influence the interpretation of muscle quality in these patients [40]. The apparent protective effect observed for neuromuscular diseases likely reflects a selection bias related to referral patterns. Patients with neuromuscular conditions are typically referred to nutrition services very early, often at diagnosis, before disease progression and prior to the onset of dysphagia. In contrast, individuals with non-neuromuscular disorders such as stroke, dementia, or cerebral palsy are usually referred due to the emergence or worsening of swallowing impairment. A previous article from our group showed that patients who entered in a protocol of fast remission to a clinical nutrition consultation had less prevalence of dysphagia than control group [41].

Across multiple studies, quadriceps echointensity and trunk intramuscular adipose tissue consistently outperform muscle thickness or radiodensity as an independent marker of swallowing impairment [40]. Other studies have shown that an increase in rectus femoris thickness is associated with dysphagia in older patients hospitalized for hip fracture; however, the etiology of dysphagia in this population differs substantially from that of patients with neurological conditions, particularly neuromuscular diseases [42]. Our multivariate analysis, controlled for sex, AI-based muscle ultrasound quality variables, and neuromuscular involvement, specifically identified sarcopenic obesity (SO) as a significant risk factor for dysphagia, further emphasizing that muscle deterioration can predict the development of swallowing. This is particularly relevant given that patients with SO in our cohort were significantly older and exhibited worse muscle quality parameters, including lower Mi and higher FATi compared to non-SO counterparts. Because these alterations lead to serious complications such as aspiration pneumonia, the identification of these specific muscle quality markers in older, sarcopenic patients with obesity is vital for enabling timely nutritional interventions and improving overall prognosis [43].

The main strength of this study is the use of AI for the evaluation of muscle ultrasonography in neurological patients. This tool allows us to reduce the variability and heterogeneity of measurements in these patients, and it also enables the standardization of muscle quality assessment through echogenicity and pennation angle. This body-composition analysis may provide new methods to monitor nutritional interventions in patients with reduced mobility, such as those with neurological conditions.

This study has several limitations. First, the heterogeneity in patient age prevents us from fully distinguishing the effects of neurological disease from those of aging in certain scenarios, although multivariate analysis can help control this confounding factor. A full nutritional assessment could not be completed for all patients, as detailed dietary intake and biochemical parameters were not collected systematically, resulting in approximately one-third missing data for these variables. These limitations reflect real-world clinical conditions, including the proportion of patients receiving tube feeding. Another limitation is the use of the Multi-Otsu algorithm to classify different components of muscle composition; these parameters have not been validated against muscle biopsy, although echogenicity may serve as a valid surrogate based on existing clinical validation studies. Although AI tools have demonstrated high technical reliability for muscle segmentation in malnutrition cohorts [14], their validation in specific neurological populations requires more rigorous scrutiny because the current evidence is limited and frequently lacks direct comparison with biological gold standards such as magnetic resonance imaging (MRI), Dual X-ray absorption (DXA) or computed tomography (CT). Another limitation of this study is that mid-arm circumference (MAMC) could not be calculated because triceps skinfold thickness was not measured systematically. A substantial selection bias exists, since most studies rely on consecutive samples from single centers or specialized clinics where patients already present a high nutritional risk, thereby producing relatively homogeneous cohorts and restricting generalizability to the broader neurological populations. Results may also be confounded by disease severity and referral patterns; for example, in amyotrophic lateral sclerosis the interval from symptom onset to nutritional assessment varies widely and influences the degree of atrophy and myosteatosis [13]. Qualitative metrics such as echogenicity indices are highly sensitive to individual factors and equipment settings, underscoring the need for multicenter studies that control these potential confounders before routine clinical integration. Lastly, no formal sample size calculation was performed due to the heterogeneity of neurological diseases, this condition limits the extrapolation of the findings, but this is an exploratory study aimed at generating new hypotheses in this patient population; the results should be interpreted cautiously and require validation in larger, adequately powered multicenter cohorts.

Future research lines could focus on developing studies across different neurological conditions using integrated parameters for the nutritional assessment of these patients. This approach would allow us to tailor population selection to specific diseases, likely at early stages, since, as shown, not all conditions share the same proportions of body-composition components. In addition, the relationship between ultrasonographic parameters and clinically relevant variables for patients, such as quality of life, dysphagia, loss of speech ability, or other complications typical of neurological disorders, could be explored. Artificial intelligence may further support this field by reducing analysis time, facilitating the identification of new radiomic biomarkers in body-composition techniques, and integrating all available data to detect diagnostic and prognostic variables related to malnutrition and to monitor nutritional treatment. Future work should prioritize longitudinal studies to track the rate of muscle quality decline and the emergence of disease-specific clusters, thereby enabling more precise, individualized medical nutrition therapy for complex neurological disorders.

## 5. Conclusions

AI-assisted ultrasonography of the rectus femoris appears to be a promising approach for assessing body composition in neurological patients at risk of malnutrition; however, these findings should be interpreted cautiously given the study’s sample and design. In our cohort, patients with neuromuscular disorders showed a lower proportion of low-echogenicity areas, consistent with preserved muscle tissue, and a higher proportion of medium- and high-echogenicity areas, which are typically associated with intramuscular fat and non-functional connective tissue. Increased medium-echogenicity (FATi) burden was associated with a higher risk of mortality, whereas AI-derived rectus femoris mass quality parameters were not associated with dysphagia. Overall, these results support the potential of AI-enhanced muscle ultrasound as a complementary tool for nutritional assessment and risk stratification in neurological populations, but external validation in larger and more diverse cohorts is required before broader generalization or clinical implementation.

## Figures and Tables

**Table 1:** Differences in nutritional assessment between sexes.

	Total (*n* = 117)	Men (*n* = 56)	Women (*n* = 61)	*p*-Value
Age (years)	63.01 (16.14)	64.73 (13.78)	61.43 (18.01)	0.27
Anthropometry				
Weight loss (%)	4.48 (0.57–12.01)	4.03 (0.83–10.59)	5.40 (−0.38–12.89)	0.57
BMI (kg/m^2^)	24.24 (5.48)	25.23 (5.31)	23.27 (5.53)	0.07
Arm circumference (cm)	26.36 (3.88)	27.43 (3.20)	25.39 (4.21)	<0.01
Calf circumference (cm)	32.78 (5.24)	33.39 (5.89)	32.21 (4.52)	0.24
Bioimpedanciometry				
Resistance (ohm)	578.58 (136.20)	514.35 (103.66)	644.12 (134.99)	<0.01
Reactance (ohm)	49.68 (13.31)	46.06 (9.85)	53.36 (15.34)	<0.01
Phase Angle (°)	4.98 (1.33)	5.12 (1.34)	4.83 (1.31)	0.28
ASMI (kg/m^2^)	6.27 (2.37)	7.57 (2.27)	4.95 (1.63)	<0.01
Rectus femoris ultrasonography (AI-assissted)				
SFT (cm)	0.94 (0.59)	0.61 (0.29)	1.25 (0.63)	<0.01
RFMT (cm)	1.08 (0.35)	1.23 (0.36)	0.95 (0.28)	<0.01
RFMA (cm)	3.32 (1.37)	3.95 (1.42)	2.73 (1.03)	<0.01
Mi (%)	44.14 (8.93)	45.33 (9.84)	43.02 (7.92)	0.16
FATi (%)	40.97 (5.57)	40.70 (6.29)	41.21 (4.86)	0.62
NMNFi (%)	14.95 (5.11)	13.97 (5.34)	15.85 (4.76)	<0.01
Pennation Angle (°)	4.87 (2.96–6.81)	5.61 (3.85–7.96)	4.08 (2.37–5.74)	<0.01
Muscle strength				
Handgrip Strength (kg)	17.18 (10.64)	21.35 (10.95)	12.64 (8.24)	<0.01
Intake assesment (*n* = 72) (Male: 38/Female:34)				
Energy Intake (kcal/kg/day)	25.49 (10.26)	21.70 (7.29)	29.74 (11.48)	<0.01
Protein Intake (kcal/kg/day)	1.19 (0.50)	0.98 (0.34)	1.43 (0.55)	<0.01
Energy Requirements Adjustment (%)	76.98 (62.30–100.99)	69.88 (55.77–90.87)	89 (67.57–120.69)	<0.01
Protein Requirements Adjustment (%)	71.79 (57.59–93.25)	61.86 (53.27–79.78)	89.41 (65.32–120.86)	<0.01
Biochemistry (*n* = 81) (Male: 40/Female: 41)				
CRP (mg/dL)	1.60 (1–4.05)	1.71 (1.09–5.08)	1.08 (1–3.52)	0.56
Albumin (g/dL)	4.42 (0.46)	4.40 (4.3–4.7)	4.35 (4.10–4.57)	0.04
Prealbumin (mg/dL)	25.29 (7.52)	25.50 (21.50–30)	24 (20.50–27.50)	0.69
CRP/Albumin	0.35 (0.23–0.89)	0.39 (0.25–1.16)	0.26 (0.23–0.73)	0.96
CRP/Prealbumin	0.06 (0.04–0.17)	0.07 (0.05–0.30)	0.05 (0.04–0.13)	0.71

BMI: Body Mass Index; ASMI: Appendicular Skeletal Muscle Index; SFT: Subcutaneous Fat Thickness; RFMT: Rectus Femoris Muscle Thickness; RFMA: Rectus Femoris Muscle Area; Mi: Low Echogenicity Index; FATi: Medium Echogenicity Index; NMNFi: High Echogenicity Index; CRP: C-reactive protein.

**Table 2:** Clinical, anthropometric, bioimpedance, ultrasonographic, muscle strength, intake assessment, and biochemical parameters according to neuromuscular involvement.

	Neuromuscular (*n* = 77)	Non neuromuscular (*n* = 40)	*p*-Value
SEX (%M/%F)	62.5/37.5	68.9/31.1	0.47
Age (years)	62.97 (15.51)	63.08 (17.49)	0.97
Weight loss (%)	5.03 (1.14–11.14)	4.09 (0.45–14.80)	0.67
BMI (kg/m^2^)	24.77 (5.49)	23.13 (5.38)	0.16
Arm circumference (cm)	26.51 (3.59)	26.07 (4.44)	0.58
Calf circumference (cm)	33.04 (5.14)	32.25 (5.45)	0.46
Resistance (ohm)	575.32 (130.34)	585.41 (149.69)	0.73
Reactance (ohm)	50.02 (14.61)	48.94 (10.25)	0.71
Phase Angle (°)	4.97 (1.36)	4.98 (1.28)	0.98
ASMI (kg/m^2^)	6.48 (2.59)	5.84 (1.77)	0.22
SFT (cm)	0.96 (0.57)	0.91 (0.65)	0.66
RFMT (cm)	1.11 (0.32)	1.04 (0.42)	0.27
RFMA (cm)	3.39 (1.23)	3.17 (1.62)	0.39
Mi (%)	42.44 (9.09)	47.36 (7.74)	<0.01
FATi (%)	41.91 (5.71)	39.15 (4.89)	0.01
NMNFi (%)	15.71 (5.03)	13.49 (4.49)	0.02
Pennation Angle (°)	5.19 (3.17–6.87)	4.36 (2.58–6.68)	0.21
Handgrip Strength (kg)	15.22 (9.33)	21.37 (12.12)	<0.01
Intake assesment (*n* = 72) (Neuromuscular: 45/Non-neuromuscular: 27)			
Energy Intake (kcal/kg/day)	26.71 (10.22)	23.48 (10.20)	0.19
Protein Intake (kcal/kg/day)	1.23 (0.45)	1.12 (0.57)	0.37
Energy Requirements Adjustment (%)	85.97 (62.67–106.30)	73.64 (62.11–88.98)	0.19
Protein Requirements Adjustment (%)	79.37 (58.15–93.14)	61.76 (44.98–98.31)	0.37
Biochemistry (*n* = 81) (Neuromuscular: 52/Non-neuromuscular: 29)			
CRP (mg/dL)	1.53 (1–4.01)	1.71 (1–5.08)	0.69
Albumin (g/dL)	4.43 (0.52)	4.42 (0.32)	0.86
Prealbumin (mg/dL)	25.18 (6.80)	25.48 (8.78)	0.86
CRP/Albumin	0.33 (0.24–0.79)	0.39 (0.23–1.16)	0.95
CRP/Prealbumin	0.05 (0.04–0.17)	0.07 (0.04–0.19)	0.92

SFT: Subcutaneous Fat Thickness; RFMT: Rectus Femoris Muscle Thickness; RFMA: Rectus Femoris Muscle Area; Mi: Low Echogenicity Index; FATi: Medium Echogenicity Index; NMNFi: High Echogenicity Index.

**Table 3:** Differences in nutritional assessment between neurological diseases in patients with direct neuromuscular involvement.

	Motorneuron Disease N = 36	Multiple Sclerosis N = 21	Parkinson Disease N = 13	Steinert’s Myotonic Dystrophy N = 7	*p*-Value
Sex (%M/%F)	58.3/41.7	61.9/38.1	69.2/30.8	71.4/28.6	0.19
Age (years)	67.75 (10.25)	48.81 (15.17)	76.69 (9.40)	55.43 (13.31)	<0.01
Weight loss (%)	4.07 (0.67–7.73)	4.44 (1.09–7.56)	12.41 (8.67–19)	0.8 (−2.90–17.58)	<0.01
BMI (kg/m^2^)	25.72 (5.77)	23.47 (4.55)	22.34 (3.27)	26.75 (7.62)	0.18
Arm circumference (cm)	26.41 (2.38)	27.37 (4.28)	24.29 (3.39)	28.48 (5.49)	<0.05
Calf circumference (cm)	33.93 (3.81)	33.60 (2.70)	30.58 (3.22)	31.21 (13.22)	0.18
Resistance (ohm)	558.83 (110.16)	591.83 (103.84)	530.42 (165.59)	694.64 (154.61)	0.04
Reactance (ohm)	46.65 (11.97)	61.17 (14.93)	43.60 (7.49)	53.07 (22.23)	<0.01
Phase Angle (°)	4.90 (1.41)	5.95 (1.22)	4.35 (0.82)	4.29 (1.07)	<0.01
ASMI (kg/m^2^)	6.89 (2.92)	6.71 (2.09)	4.76 (1.68)	6.73 (2.37)	0.12
SFT (cm)	0.87 (0.51)	0.98 (0.43)	0.87 (0.47)	1.54 (1.02)	0.03
RFMT (cm)	1.12 (0.35)	1.19 (0.29)	0.95 (0.20)	1.14 (0.31)	0.23
RFMA (cm)	3.52 (1.41)	3.64 (1.12)	2.91 (0.96)	2.90 (0.79)	0.18
Mi (%)	40.29 (6.83)	49.17 (10.59)	40.71 (6.89)	35.57 (8.03)	<0.01
FATi (%)	43.64 (4.75)	37.39 (6.18)	43.03 (4.37)	44.45 (3.89)	<0.01
NMNFi (%)	16.07 (4.39)	13.44 (6.57)	16.26 (4.08)	19.62 (4.97)	0.04
Pennation Angle (°)	4.93 (3.73–6.52)	5.07 (2.12–6.96)	6.37 (3.25–7.87)	5.99 (2.57–8.47)	0.54
Handgrip Strength (kg)	13.27 (8.99)	21.5 (9.85)	14.58 (7.38)	9.5 (5.36)	0.01
INTAKE ASSESMENT (*n* = 45) (MD: 22; MS: 11; PD: 10; SMD: 2)					
Energy Intake (kcal/kg/day)	24.11 (7.87)	26.29 (8.56)	34.99 (12.78)	16.21 (5.95)	0.01
Protein Intake (kcal/kg/day)	1.17 (0.36)	1.17 (0.32)	1.55 (0.62)	0.69 (0.30)	0.03
Energy Requirements Adjustment (%)	80.36 (26.24)	87.63 (28.54)	116.65 (42.61)	54.03 (19.85)	0.01
Protein Requirements Adjustment (%)	77.79 (24.25)	78.32 (21.28)	103.61 (41.46)	46.34 (20.00)	0.03
BIOCHEMISTRY (*n* = 52) (MD: 25; MS: 13; PD: 7; SMD: 5)					
CRP (mg/dL)	1.57 (1–3.27)	1.17 (1–5.69)	1.63 (1–37.59)	1.88 (1.04–4.58)	0.06
Albumin (g/dL)	4.53 (4.40)	4.54 (0.27)	4.14 (0.31)	4.10 (0.19)	0.07
Prealbumin (mg/dL)	24.41 (8.08)	27.61 (5.60)	23.87 (5.49)	24.83 (4.45)	0.53
CRP/Albumin	0.32 (0.23–0.74)	0.27 (0.22–1.33	0.33 (0.24–0.53)	0.37 (0.26–0.89)	0.44
CRP/Prealbumin	0.06 (0.04–0.17)	0.05 (0.04–0.17)	0.06 (0.04–0.07)	0.06 (0.05–0.21)	0.49

SFT: Subcutaneous Fat Thickness; RFMT: Rectus Femoris Muscle Thickness; RFMA: Rectus Femoris Muscle Area; Mi: Low Echogenicity Index; FATi: Medium Echogenicity Index; NMNFi: High Echogenicity Index; MD: Motorneuron Disease; MS: Multiple Sclerosis; PD: Parkinson Disease; SMD: Steinert’s Myotonic Dystrophy.

**Table 4:** Correlation between AI-based ultrasound variables against classical muscle and function variables.

	BMI	Calf Circumference	Resistance	Reactance	Phase Angle	Handgrip Strength
Mi	r = −0.11; *p* = 0.92	r = 0.09; *p* = 0.40	r = −0.09; *p* = 0.35	r = 0.16; *p* = 0.11	r = 0.24; *p* = 0.02	r = 0.27; *p* = 0.01
FATi	r = 0.10; *p* = 0.31	r = −0.09; *p* = 0.33	r = −0.16; *p* = 0.11	r = −0.12; *p* = 026	r = −0.11; *p* = 0.31	r = −2,01; *p* = 0.05
NMNFi	r = 0.07; *p* = 0.50	r = −0.04; *p* = 0.71	r = −0.14; *p* = 0.16	r = −0.14; *p* = 0.16	r = −0.28; *p* < 0.05	r = −0.23; *p* = 0.03
Pennation Angle	r = 0.08; *p* = 0.46	r = 0.07; *p* = 0.51	r = −0.23; *p* = 0.02	r = −0.03; *p* = 0.79	r = 0.16; *p* = 0.11	r = 0.23; *p* = 0.03
SFT	r = 0.28; *p* < 0.01	r = 0.19; *p* = 0.06	r = 0.29; *p* < 0.01	r = 0.34; *p* < 0.01	r = 0.06; *p* = 0.56	r = −0.29; *p* < 0.01
RFMA	r = 0.30; *p* < 0.01	r = 0.29; *p* = 0.04	r = −0.23; *p* < 0.01	r = 0.18; *p* = 0.08	r = 0.53; *p* < 0.01	r = 0.51; *p* < 0.01
RFMT	r = 0.27; *p* < 0.01	r = 0.35; *p* < 0.01	r = −0.45; *p* < 0.01	r = 0.12; *p* = 0.24	r = 0.54; *p* < 0.01	r = 0.49; *p* < 0.01

BMI: Body Mass Index; SFT: Subcutaneous Fat Thickness; RFMT: Rectus Femoris Muscle Thickness; RFMA: Rectus Femoris Muscle Area; Mi: Low Echogenicity Index; FATi: Medium Echogenicity Index; NMNFi: High Echogenicity Index.

**Table 5:** Differences in Nutritional Assessment variables between patients who died and survivors.

Exitus	Yes (*n* = 26)	No (*n* = 91)	*p*-Value
Sex (%H/%M)	38.5/61.5	50.5/49.5	0.28
Age (years)	71.38 (10.10)	60.62 (16.78)	<0.01
ANTHROPOMETRY			
Weight loss (%)	8.25 (4.18–17.07)	3.75 (0.43–10.13)	0.02
BMI (kg/m^2^)	24.40 (3.09)	24.18 (6.10)	0.86
Arm circumference (cm)	25.06 (2.64)	26.75 (4.11)	0.05
Calf circumference (cm)	32.99 (2.84)	32.72 (5.62)	0.81
ELECTRICAL BIOIMPEDANCIOMETRY			
Resistance (ohm)	559.44 (157.16)	585.04 (128.91)	0.42
Reactance (ohm)	46.98 (12.72)	50.59 (13.47)	0.24
Phase Angle (°)	4.69 (1.19)	5.07 (1.36)	0.22
ASMI (kg/m^2^)	5.40 (1.49)	6.56 (2.54)	0.04
RECTUS FEMORIS ULTRASONOGRAPHY (AI-ASSISSTED)			
SFT (cm)	0.91 (0.50)	0.95 (0.62)	0.73
RFMT (cm)	1.01 (0.29)	1.11 (0.37)	0.19
RFMA (cm)	3.17 (1.14)	3.36 (1.44)	0.54
Mi (%)	40.91 (5.81)	45.07 (9.47)	0.04
FATi (%)	43.03 (4.05)	40.37 (5.82)	0.03
NMNFi (%)	16.05 (3.12)	14.63 (5.52)	0.21
Pennation Angle (°)	4.68 (3.09–6.41)	5.03 (2.81–7.24)	0.69
MUSCLE STRENGTH			
Handgrip Strength (kg)	11.50 (8.19)	19.13 (10.73)	<0.01
INTAKE ASSESMENT (*n* = 72) (Yes: 16; No: 56)			
Energy Intake (kcal/kg/day)	30.14 (10.34)	24.17 (9.94)	0.05
Protein Intake (kcal/kg/day)	1.39 (0.48)	1.14 (0.49)	0.08
Energy Requirements Adjustment (%)	100.48 (34.46)	80.57 (33.14)	0.05
Protein Requirements Adjustment (%)	92.50 (32.03)	75.81 (33.21)	0.08
BIOCHEMISTRY (*n* = 81) (Yes: 19; No: 62)			
CRP (mg/dL)	1.28 (1–4.84)	1.63 (1–4.04)	0.92
Albumin (g/dL)	4.35 (0.32)	4.46 (0.49)	0.32
Prealbumin (mg/dL)	24.21 (5.20)	25.62 (8.10)	0.48
CRP/Albumin	0.29 (0.24–1.04)	0.36 (0.23–0.85)	0.41
CRP/Prealbumin	0.05 (0.04–0.21)	0.06 (0.04–0.17)	0.38

SFT: Subcutaneous Fat Thickness; RFMT: Rectus Femoris Muscle Thickness; RFMA: Rectus Femoris Muscle Area; Mi: Low Echogenicity Index; FATi: Medium Echogenicity Index; NMNFi: High Echogenicity Index.

## Data Availability

Data are not available due to ethics restrictions of the organization.
